# Boosting the Electrocatalytic CO_2_ Reduction Reaction by Nanostructured Metal Materials via Defects Engineering

**DOI:** 10.3390/nano12142389

**Published:** 2022-07-13

**Authors:** Shuangyang Zhao, Aihua Liu, Yonghe Li, Yanyan Wen, Xiaoqian Gao, Qiaoli Chen

**Affiliations:** State Key Laboratory Breeding Base of Green Chemistry Synthesis Technology, College of Chemical Engineering, Zhejiang University of Technology, Hangzhou 310014, China; zshuangyang@outlook.com (S.Z.); aihualiu@zjut.edu.cn (A.L.); yongheli@zjut.edu.cn (Y.L.); 2112101442@zjut.edu.cn (Y.W.); 2111901044@zjut.edu.cn (X.G.)

**Keywords:** CO_2_ reduction reaction, defects, electrocatalytic, metal, nanomaterials

## Abstract

Electrocatalytic CO_2_ reduction reaction (CO_2_RR) is one of the most effective methods to convert CO_2_ into useful fuels. Introducing defects into metal nanostructures can effectively improve the catalytic activity and selectivity towards CO_2_RR. This review provides the recent progress on the use of metal nanomaterials with defects towards electrochemical CO_2_RR and defects engineering methods. Accompanying these ideas, we introduce the structure of defects characterized by electron microscopy techniques as the characterization and analysis of defects are relatively difficult. Subsequently, we present the intrinsic mechanism of how the defects affect CO_2_RR performance. Finally, to promote a wide and deep study in this field, the perspectives and challenges concerning defects engineering in metal nanomaterials towards CO_2_RR are put forward.

## 1. Introduction

The fast development of society and industrial activities requires massive primary energy resources of fossil fuels, leading to the continuously fast increase of CO_2_ emissions [1], accompanied with the severe global climate change and environmental issues. The effective CO_2_ capture and utilization is very important for a sustainable and clean development [2,3,4]. CO_2_ is a thermodynamically stable molecule with the carbon atom in the fully oxidized state. It takes much energy to break the molecule due to its linear symmetric feature from containing two equivalent carbon–oxygen double bonds of CO_2_ [5]. The key to activate the conversion of CO_2_ into fuels is to fully make use of the empty orbital of the CO_2_ molecule to accept electrons and produce reduced carbon species, although it is a difficult process due to the poor kinetics of CO_2_ reduction and a low desired products selectivity [6].

So far, conversions based on thermochemical pathway [7], photochemical pathway [8,9], electrochemical pathway [10], photoelectrochemical pathway [11] and photothermal pathway [12,13] have been widely developed to convert CO_2_ to valuable fuels and chemicals. Among them, the electrocatalytic CO_2_ reduction reaction (CO_2_RR) remains one of the most effective ways and has especially attracted attention as it has several advantages as follows. Firstly, the reaction can be conducted at ambient mild conditions without a high temperature or high pressure. Secondly, the reaction rate could be easily controlled by tuning the external bias (i.e., overpotential), which indirectly solve the problem of intermittency and redundancy of electricity generated by wind power and waterpower. Last but not least, the products could be varied according to the electrodes loaded with different catalysts [10,14].

Metals nanomaterials, especially noble metals nanomaterials, have been widely studied as electrocatalysts towards many important electroreactions, such as the hydrogen evolution reaction (HER), oxygen reduction reaction (ORR) and nitrogen reduction reaction (NRR), because of their excellent conductivity, high catalytic activity and stability under electrochemical conditions. For example, Pt–based nanomaterials rank as the state–of–the–art catalysts in fuel cells [15]. Pd nanomaterials achieve the highest catalytic performance towards HER [16]. As for CO_2_RR, Cu catalysts are most studied because they facilitate the formation of C_2+_ products, which are the products that contain multiple carbon atoms in the molecular formula. Nowadays, the C_2+_ products from CO_2_RR include C_2_ compounds of C_2_H_4_, C_2_H_5_OH, CH_3_CHO, CH_3_COOH, HOOCCOOH, and C_3_ compounds of C_3_H_6_, C_3_H_7_OH and C_2_H_5_CHO [17]. Moreover, Pd, Au, Ag, Sn, Bi and their alloys are widely explored for achieving C_1_ products of CO, CH_4_, HCOOH, HCHO and CH_3_OH with high conversion efficiency and selectivity [18,19,20,21,22,23].

To obtain a higher activity and a high usage efficiency of metal, great efforts have been devoted to manipulating the metal nanomaterials, including the morphology, surface, composition and crystal phase. The intrinsic factor could be ascribed to the theories concerning the optimization of electronic structure to achieve the proper adsorption of reaction intermediates for the highest activity. With the rapid development of nanomaterials synthesis methods and the characterization techniques that deepen into the atomic level, the defect structures modulation of metal nanomaterials has attracted much attention and yielded great results. The introduction of defects into nanomaterials, as an effective strategy to greatly change the surface structure, charge distribution of the catalysts and expose more catalytic active sites due to the high distortion energy and diverse atomic rearrangements, plays a crucial role in improving the activity of catalytic reactions. Nowadays, the applications of catalysts with rich defects, showing better electrocatalytic performance than the pristine catalysts, have been summarized in reactions of NRR, ORR, oxygen evolution reaction and HER [24,25,26,27]. To make further great progress on CO_2_RR, a summary and introduction of the corresponding structure features, synthesis methodology and the characterization technique are urgently required as well.

This review aims to summarize the defect types in metal nanomaterials, by introducing structure characterizations of defects by electron microscopy (EM). Moreover, recent advances in the systematic design and creation of defects in metal nanomaterials with controllable defects that focus on CO_2_RR are summarized. Finally, we present some perspectives on the design of nanostructured metal electrocatalysts for useful fuels production by electrochemical CO_2_RR.

## 2. Defects in Metal Nanomaterials

**Figure 1:**
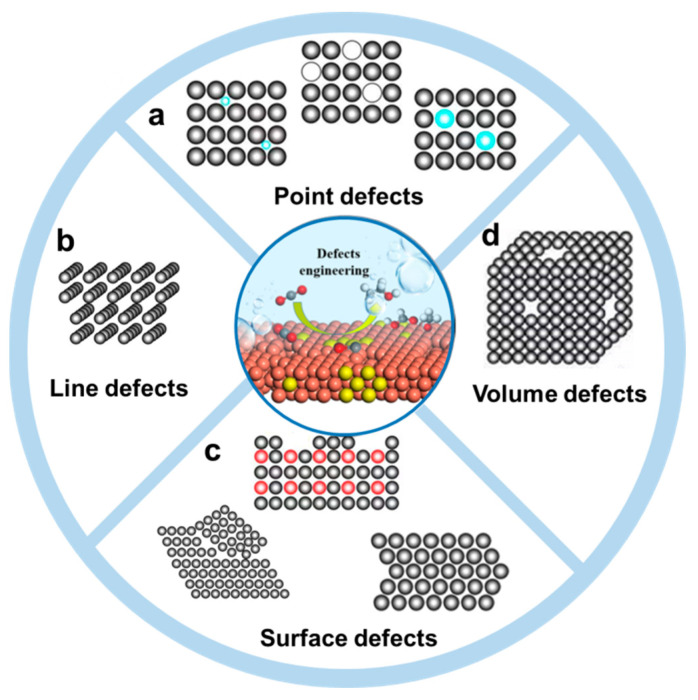
Schematic illustration of various defects in metal nanomaterials. (**a**) Point defects, (**b**) line defects, (**c**) surface defects and (**d**) volume defects.

Crystals have periodic structures while the appearance of defects means a deviation from that periodic structure. Depending on the different fields that use defects, the classification of defects could be in different forms. For example, the defects of crystal can be classified into four types, zero–dimensional defects (point defects), one–dimensional defects (line defects, known as dislocation), two–dimensional defects (surface defects) and three dimensional defects (volume defects), according to their dimensionality [28]. On the other hand, according to the position of defects in crystals, defects can be classified into bulk defects and surface defects, which further includes point defect, line defect and plane defect. According to the generation atoms of defects, defects can be divided into intrinsic defects and doped defects. Furthermore, different materials have different types and features of defects, such as metal organic frameworks (MOFs), carbon–based materials, metal materials and transition metal compounds [29]. Based on the definition and structural features of metal nanomaterials, the defects in metal nanomaterials are classified into four distinct types, including point defects, line defects, surface defects and volume defects, which are displayed in Figure 1.

### 2.1. Point Defects

**Figure 2:**
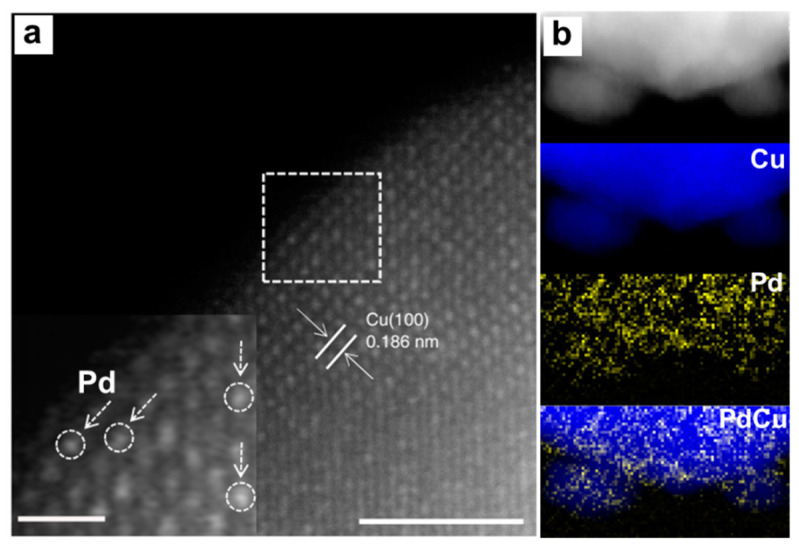
Structural characterization of point defects in Pd−doped Cu catalysts. (**a**) HAADF−STEM image and inverse FFT pattern (inset) of selected area. Scale bars are 2 nm. (**b**) EELS mapping. Reproduced with permission [36]. Copyright 2016, Springer Nature.

The point defects are related to the loss, replacement of a normal atomic lattice point, or occupation of a gap position (Figure 1a), thus creating localized disruptions in the crystal structure. In metal nanomaterials, vacancy defects and doping defects are the main forms of point defects [30,31]. Doping defects mean the replacement of the intrinsic atomic lattice by other metal atoms or nonmetal atoms. A vacancy refers to the removal of neutral atoms in the lattice of metals without breaking the electrical equilibrium of the whole structures. These defects are so small in size and numbers that direct observation is difficult. With much effort devoting to the pursuit of higher spatial resolution, EM techniques allow the direct atomic–scale observation of the structure [32,33,34,35]. Thus, the defects in various materials, including the materials containing the light elements, such as carbon materials, MOF and covalent organic framework, could be revealed. As the Z contrast in scanning transmission electron microscope (STEM) image depends on the atomic number, the doping and vacancy defects could be confirmed based on their Z−contrast distinction. For example, the Pd−doped Cu electrocatalysts were analyzed by high–angle annular dark–field scanning transmission electron microscopy (HAADF−STEM) imaging (Figure 2a) [36]. Due to the Z–value of Pd being higher than that of Cu, the bringer atomic site indicated the individual Pd atom in the Pd–doped Cu catalysts. The inverse fast Fourier transition (FFT) pattern further confirmed the doping of Pd atoms in replacement of Cu atoms. On the other hand, high–resolution electron energy–loss spectroscopy (EELS) mapping indicated that the doped Pd atoms were distributed evenly in the Cu catalysts (Figure 2b). Recently, Zhang and co–workers reported a wet chemical synthesis of hierarchical Rh nanostructures, which were composed of ultrathin nanosheets with ordered vacancies and a small amount of interstitial carbon [37]. Due to the unique defects in structure that facilitated the adsorption and dissociation of H_2_O, the obtained Rh nanostructures exhibited a remarkably enhanced electrocatalytic activity and stability toward HER in alkaline media. To obtain the defects information, the HAADF–STEM images together with structure fittings were taken to analyze the structure of the Rh nanostructures, which showed two types of ordered vacated defects. Due to the large number of defects, the crystal phases were changed.

### 2.2. Line Defects

**Figure 3:**
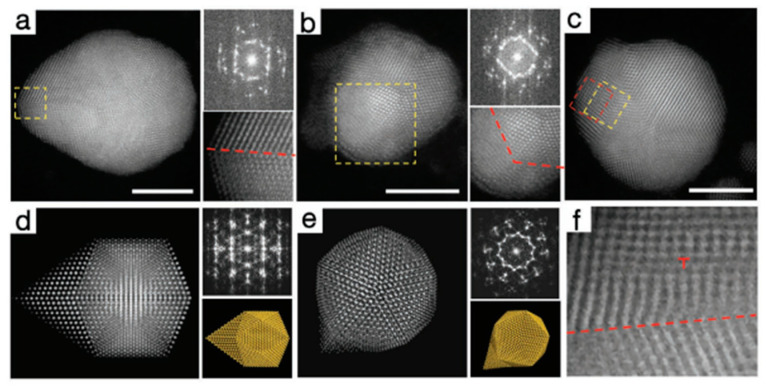
Structural characterization of line defects in symmetry−breaking Au icosahedral catalysts. HAADF−STEM images, FFT patterns and magnified image of the selected area projected close to (**a**) [112] and (**b**) [110] direction. (**c**) HAADF–STEM image of an initial Au structure. STEM image simulation, FFT patterns and atomic structure models projected along (**d**) [112] and (**e**) [110] directions. (**f**) Enlarged view of the selected area in (**c**). Scale bars are 5 nm. Reproduced with permission [38]. Copyright 2020, Wiley–VCH.

Line defects, which can be classified into screw dislocations, edge dislocations and mix dislocations, are deformations caused by atomic planes sliding over each other. In metal nanomaterials, dislocations are always formed during the formation process of nanomaterials. Zhu et al. reported the wet chemical synthesis of symmetry–breaking Au icosahedral nanocrystals, which exhibited a high activity toward photocatalytic ammonia borane hydrolysis [38]. The growth process was investigated by the HAADF–STEM. It was found that the symmetry–breaking structure started from a five–twinned icosahedron and grew a twinned protruding island. Moreover, it was observed that an edge dislocation occurred in the vicinity of the twin boundary at the initial growth process of the symmetry−breaking structure (Figure 3c,f).

Thus, a structural model composed of a {111}–faceted icosahedron capped with {100}–faceted twin–tetrahedral units was proposed and further validated by matching the experimental high–resolution STEM imaging, FFT patterns and their simulated ones along the [112] and [110] directions, respectively (Figure 3a,b,d,e). From the viewpoint of microscopic growth, they proposed the formation of symmetry–breaking Au nanocrystals could be ascribed to the site–specific nucleation of stacking faults and dislocations based on the seeded growth method.

### 2.3. Surface Defects

**Figure 4:**
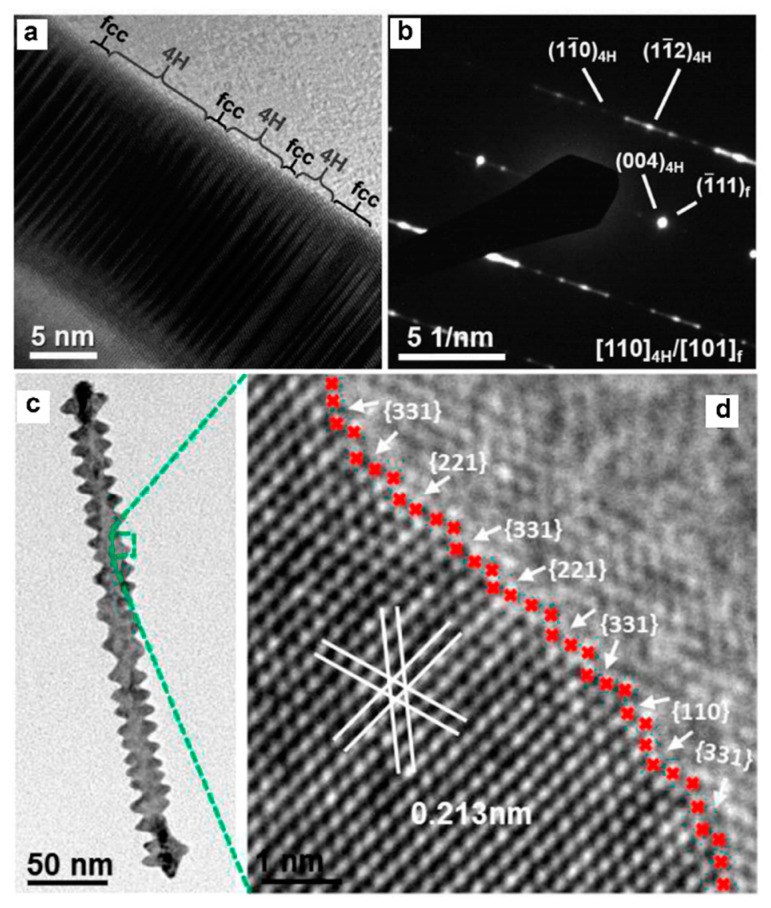
Structural characterization of surface defects in (**a**,**b**) 4H/fcc Au nanorod and (**c**,**d**) Pt–Cu nanowire. (**a**) HRTEM image showing alternating 4H and fcc crystal phases. (**b**) SAED pattern from zone axes of [110]_4H_/[101]_f_. Reproduced with permission [52]. Copyright 2017, Wiley–VCH. (**c**) TEM and (**d**) HRTEM images of an individual nanowire. Reproduced with permission [53]. Copyright 2016, American Chemical Society.

Surface defects include surface steps, stacking faults, grain boundaries and twin boundaries. In metal nanomaterials, these four surface defects widely exist. Among them, twin boundaries could be viewed clearly in five–twinned structures, including decahedron, icosahedron and twinned nanorods [39,40,41,42]. Grain boundaries are the interfaces that usually separate a large number of randomly oriented grains while each grain is a single crystal. To characterize the twin boundaries, electron diffraction is usually used apart from the EM images. Taking the electron diffraction of five–twinned structure as an example, the pattern contains an interpenetrated set of five individual diffraction patterns along the [110] direction (e.g., Figure 3b,e) [38,43,44,45,46]. One set is assigned to the [110] zone axis, and others could be obtained by rotating the former one by ca. 72°. A stacking fault is a region in the crystal where the regular stacking sequence is interrupted. For example, the metal with face–centered cubic (fcc) phase has a periodic arrangement of atomic sheets in the form of ABCABC… The presence of a stacking sequence AB or ABCB instead of ABC could be regarded as a stacking fault. However, a large area of stacking sequence ABCB or AB leads to phase changes. From the transmission electron microscope (TEM) image, there are stripes with different contrast at relatively low magnification. To further analyze the stacking faults, the sequence of atomic arrangements should be obtained from a high−resolution transmission electron microscope (HRTEM) image or STEM image. One−dimensional nanowires or nanorods usually have many stacking faults in structures [47,48,49,50,51]. For example, Zhang et al. synthesized Au nanorods with alternating 4H/fcc crystal–phase heterostructures [52]. The corresponding structure feature and analysis is shown in Figure 4. A large number of stripes are seen in the TEM image (Figure 4a). The stacking sequence ABCBABCB…. was obtained, which could be ascribed to the 4H phase. The selected–area electron diffraction (SAED) further confirmed the mixture structure (Figure 4b) because more than one set of points could be found. A careful analysis shows these points could be told apart to be the characteristic [101]_4H_ and [110]_f_ zone diffraction patterns of the 4H and fcc phases, respectively.

A step is an essential kind of surface defect in metal electrocatalysts, and it influences the surface atom coordination number, which is closely connected to the surface electronic structure and catalytic performance [54,55,56]. To view the surface steps, a specific direction should be projected by electron beam. For example, Huang et al. reported an effective approach to synthesize screw–thread–like Pt–Cu alloy nanowires that combined the advantages of a high surface area and high–index facets, showing boosted electrocatalytic performance [53]. Pt–Cu nanowires are single crystalline. When the [110] direction was projected, the steps on the high−index facets of {110}, {221}, and {331} were observed (Figure 4d).

### 2.4. Volume Defects

**Figure 5:**
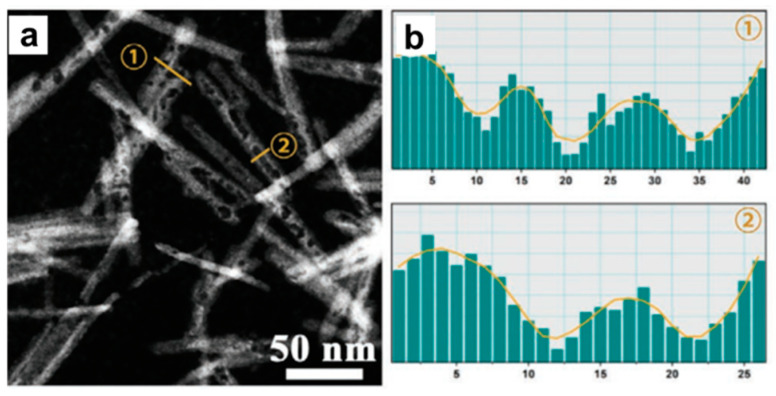
Structural characterization of volume defects in Cu nanoribbons. (**a**) HAADF–STEM images. (**b**) The corresponding intensity profile along the lines that marked by numbers 1 and 2 with yellow color in (**a**). Reproduced with permission [57]. Copyright 2021, Wiley–VCH.

Volume defects are crystal defects in three–dimensional space, which include pores, cracks, foreign inclusions and other phases that are normally introduced during processing and fabrication steps. Porous structures, the main form of metal volume defect, are featured by a large number of pore structures inside them. The pore structure can be clearly seen in HAADF–STEM images, due to the porous sites having a darker contrast because of the fewer atoms. For example, Yang et al. reported the construction of mesoporous Cu nanoribbons by in situ electrochemical reduction of Cu–based metal organic frameworks [57]. The HAADF–STEM image revealed that a high density of mesopores was distributed throughout the whole of nanoribbons (Figure 5a) as there were many dark pores on the bright Cu nanoribbons. The information could be further extracted from the HAADF–STEM image, which clearly showed the appearance of pores. For example, the intensity profile in a HAADF–STEM image was extracted to confirm the pores (Figure 5b). The multiple ups and downs of the intensity indicate the existence of mesopores. To further gain more information of pore size distribution, the N_2_ adsorption−desorption could be measured.

## 3. Relationship between Defects and CO_2_RR

**Figure 6:**
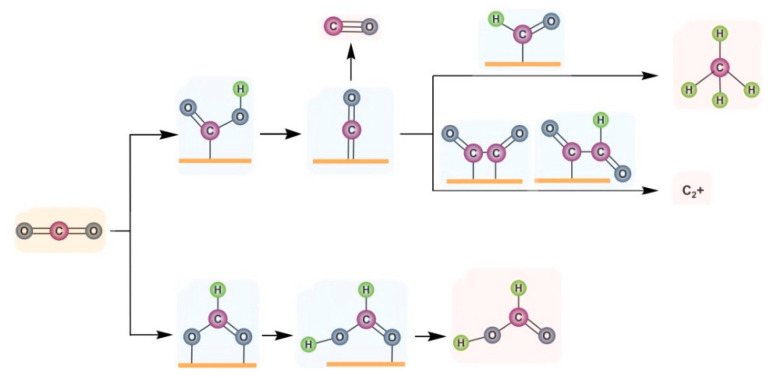
Schematic mechanism for producing different products in CO_2_RR from *COOH and *OCHO intermediates, respectively.

The electrocatalytic reduction of CO_2_ is a complex process, involving electron transfer reactions of 2–12. According to the different number of electron transfer, the generated products generally include CO, CH_3_OH, HCHO, CH_4_, HCOOH, C_2_H_4_, C_2_H_5_OH, CH_3_COOH and so on. In addition, as a two−electron reaction process, HER is a competitive reaction for electrocatalytic CO_2_RR. The CO_2_RR process is generally divided into three stages: the reactant CO_2_ molecules diffuse in the liquid phase and adsorb on the surface of catalysts at the three–phase interface. Through electron transfer and multistep protonation, different reaction intermediates are formed. Then, the intermediates are eventually reduced to products and then desorb and diffuse outward into the electrolyte. As shown in Figure 6, after the adsorption and activation of CO_2_ molecules, the intermediates *COOH and *OCHO are formed through electron–proton coupling. For the *COOH intermediate, a proton attack occurs on the specific oxygen atom, resulting in the break of the C–O bond to produce the *CO intermediate. The conversion from *COOH to *CO is a thermodynamically downhill process. For weakly adsorbed *CO, *CO is easy to desorb from the surface, and then it forms the product CO. Catalysts, such as Zn, Au and Ag, could selectively generate *COOH intermediates and have weak adsorption for *CO, leading to the main product CO. The moderate adsorption strength of *CO is favorable for subsequent protonation to form HCHO, CH_4_, CH_3_OH or C_2+_ products. Taking CH_4_ as an example (Figure 6), after obtaining the adsorbed *CO intermediate, hydrogenation reactions then take place to produce *CHO, further *CH_2_O and *OCH_3_ intermediates, and finally CH_4_. Through the dimerization of *CO and electron transfer to form *OCCO intermediates, a further protonation to get *OCCOH followed by a reduction, C_2_H_4_ and other C_2_ products could be produced. For generation products such as CH_3_CH_2_CH_2_OH, multiple rate control steps are required, leading to a low catalytic activity and selectivity. For the *OCHO intermediate, which is formed by a proton attack on the carbon atom, a further hydrogenation occurs to produce HCOO^−^ or HCOOH. At present, the selectivity of CO or HCOOH in C_1_ products can reach more than 90% [58,59], while the selectivity of methane is around 80% [60]. Moreover, the selectivity of C_2_ products (e.g., ethylene) can reach 80% [61].

The electronic structure has been proved to be critical to enhance the activity and durability. Defects engineering is an important strategy to significantly manipulate the surface electronic structure of electrocatalysts. The defect concentration of metal causes the change of the electronic band structure and local charge distribution of the electrocatalysts, thus finally improving their activity and selectivity. Catalytic reactions often occur on the surface or interface of metal materials, resulting in the more important role of surface defects than bulk defects.

**Figure 7:**
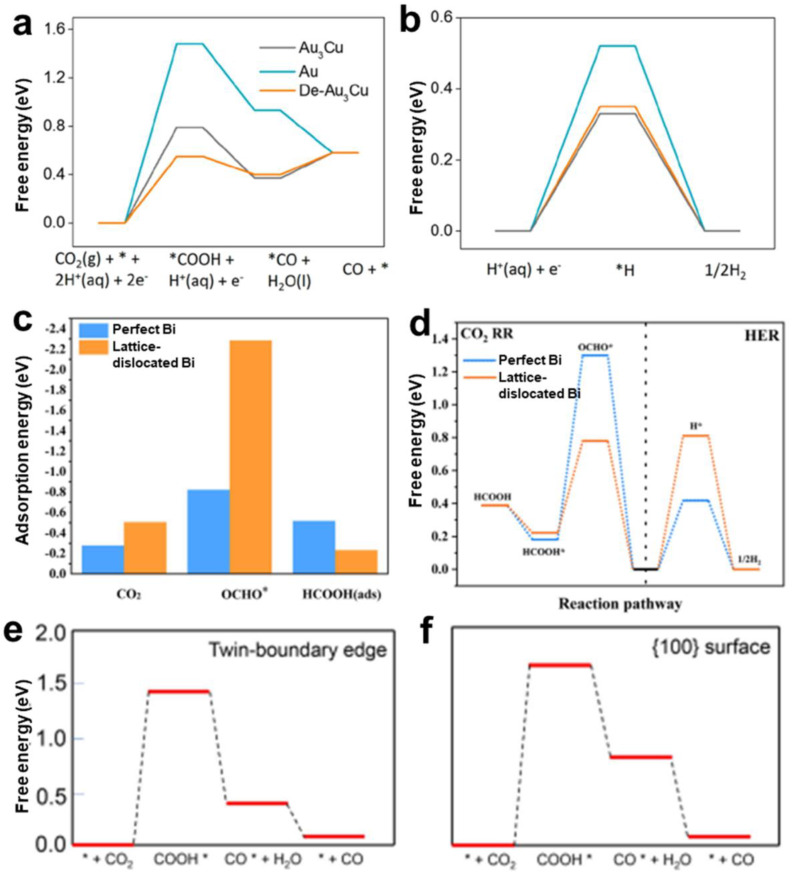
Relationship between defects and CO_2_RR. Free energy of Au, Au_3_Cu and dealloyed Au_3_Cu (De–Au_3_Cu) for the generation of (**a**) CO and (**b**) H_2_. Reproduced with permission [31]. Copyright 2018, American Chemical Society. (**c**) Adsorption energy of CO_2_, OCHO* and HCOOH (ads). (**d**) Free energies plots of HCOOH and H_2_ formation for perfect and lattice–dislocated Bi nanosheets. Reproduced with permission [64]. Copyright 2022, Elsevier. Free energy plots for CO_2_RR at the (**e**) twin−boundary edge and (**f**) on the Ag {100} surface. Reproduced with permission [68]. Copyright 2020, Elsevier.

Point defects display localized disruptions in crystal structure and have been widely investigated in CO_2_RR [31,62]. The electronic structure of metal materials with point defects is connected with the novel features derived from the redistributions of the spin and charge, which is accompanied by the improvement in catalytic properties. As has been reported, the changing electronic structure is closely connected with the adsorption energy of intermediates, which might markedly affect the selection of a reaction pathway. The vacancy and low–coordination sites in metal materials with vacancy defects dedicate much to the capture of electrons and the increase of highly selective sites. For instance, the formation of Cu vacancies on the cubic AuCu catalysts via dealloying contributes to a decrease of overpotential for CO products, an enhancement of CO selectivity (the maximum is 94.3%) and mass current density [31]. As mentioned in Figure 6, *COOH and *CO are key intermediates for CO formation and the conversion from *COOH to *CO is an energy−favorable process; the balance of *COOH adsorption and CO desorption should be taken into consideration. Density functional theory (DFT) calculations have indicated that the energy barrier (free energy shown in Figure 7a) for *COOH adsorption of dealloyed Au_3_Cu is lower than that of perfect Au_3_Cu and Au. However, the energy barrier for *CO desorption and *H adsorption (Figure 7b) is quite close to each other. Thus, a balance of *COOH adsorption and CO desorption was achieved on the defect sites on dealloyed Au_3_Cu, leading to the most optimal CO selectivity. Doping means the replacement of one atom by another atom or sometimes more than one kind of atoms, which is called co–doping. New bonds are generated during the doping process. The combination of doped heteroatoms alters the electronic structure and further impacts the adsorption and desorption behaviors of key intermediates, which mainly influences the pathway of CO_2_RR. For example, Li et al. reported a metal doping approach to modulate the adsorption of hydrogen, which facilitated the hydrogenation of C_2_ intermediates (HOCCH*) and thus improved the faraday efficiency (FE) for alcohols, while suppressing the formation of ethylene [36]. Chen et al. used the doping of nonmetal B to induce the formation of electron–rich Bi and adjusted the local electron density of the active center, thus optimizing the adsorption energy of *OCHO intermediates and facilitating the formation of formates [63].

Lattice dislocations, which can cause compressive/tensile deformation around corresponding fields, have been clarified to enhance the conductivity, create more active sites and adjust the adsorption ability of reactants and intermediates. Wang et al. prepared ultrathin Bi nanosheets with lattice dislocations that exhibited superior activity, selectivity and durability towards CO_2_RR with selective HCOOH production [64]. Bi (101) surface and that with 10% uniaxial expansion were constructed for perfect Bi and lattice–dislocated Bi nanosheets to calculate the adsorption energy and free energy of reaction pathway, respectively. The higher negative adsorption energy of CO_2_ and OCHO*, and the lower negative adsorption energy of HCOOH on lattice–dislocated Bi (Figure 7c) indicated that lattice–dislocated Bi was more favorable for the adsorption of CO_2_ and OCHO*and desorption of HCOOH than perfect Bi, thus promoting the high selectivity of HCOOH. Meanwhile, compared with lattice–perfect Bi, there was a lower formation barrier of OCHO* (the vital intermediate in the production of HCOOH) and a higher barrier of hydrogen for lattice–dislocated Bi (Figure 7d), further indicating its advantage on HCOOH formation and disadvantage on the formation of H_2_. In conclusion, the construction of lattice dislocations contributed to the rearrangement of the electric structure and optimized the adsorption conditions of intermediates.

Grain boundaries and twin boundaries are commonly considered to be active over CO_2_RR. Grain boundaries have been proven to be active sites in the promotion of the selectivity of products, since the study by Li et al. revealing the linear relationship between grain boundaries and performance of CO electroreduction reaction [65]. It has been revealed by calculations that there are abundant sites on grain boundaries or twin boundaries of Au nanoparticles. Among them, a considerable fraction (10%) of sites could markedly break the scaling relationship of formation energy of *COOH and binding energy of *CO [66]. A DFT study also revealed the outstanding role that the grain boundaries sites of a specific facet play in the CO_2_RR of Au catalysts to change the reaction route. Different types of grain boundaries could behave very differently [67]. On the grain boundaries sites of Au (100) facet, the barrier for the formation of *COOH from CO_2_ is the potential determination step while the release of CO is an exergonic process. Thus, this kind of grain boundary could lower the overpotential of gaseous CO rather than change the pathway. On the other hand, the potential determination step is the formation of *CHO from *CO because of the strong binding with CO on grain boundaries sites of Au (110) facet, resulting in the pathway change of CO_2_RR. Furthermore, the catalytic CO_2_RR activity resulting from atoms on {100} facets, single crystal edges and twin boundary edges have been quantitatively evaluated through calculating the atom−specific activity (i.e., average current generated from one atom) over Ag nanocubes and five twinned Ag nanowires, which both have {100} facets exposed, but showing different types of edges [68]. The catalytic activity of atoms on twin boundary edges is similar to that on single crystal edges, but more than two orders higher than that on {100} facets. DFT calculations of free energy of the intermediates on twin boundary edge (Figure 7e) and the {100} surface (Figure 7f) revealed that the intermediate COOH* stabilized via a bridge−type binding is easier to form at twin boundary than that on the {100} surface, thus being favorable to a higher activity for the conversion of CO_2_ to CO.

Volume defects can maximize the active sites and optimize the local pH. The catalysts of the porous materials with volume defects have a high specific surface area, which is very useful for CO_2_ adsorption. Moreover, a relatively high density of active sites can be endowed in these materials, which can improve the efficiency of energy conversion. Different catalytic effects can be obtained by adjusting the pore size and pore density. Porous materials can also react in severe environments without significantly reducing the porosity of the materials. It has been found that porous metals are promising catalysts for electrocatalytic CO_2_ reduction. Highly porous structure in Zn catalyst caused a high local pH which suppressed H_2_ evolution, resulting in a boosted CO selectivity [69]. On the other hand, the introduction of new phases is one kind of volume defects. The generation of new phases is conducive to the reconstruction of the intrinsic electronic structures and provides original active sites for the CO_2_RR process. Chen et al. [70] reported a rational approach for preparing heterostructured intermetallic CuSn catalysts (Cu_3_Sn/Cu_6_Sn_5_), which showed a high efficiency of 82% for the production of formate at −1.0 V vs. reversible hydrogen electrode (RHE), while the Cu_6_Sn_5_ and Cu_3_Sn catalysts mainly produced H_2_, showing 75.3% and 57.5% at the same potential, respectively. The DFT calculation results revealed that the Cu_3_Sn/Cu_6_Sn_5_ was preferred for the generation of formate, as well as the experimental results. The calculated adsorption energies of HCOO* were more negative than that of *COOH, and the energy difference between HCOO* and *H was beneficial for a high selectivity of the formate.

## 4. Defects Engineering

So far, a variety of methods are capable of creating defects. In general, these methods to construct defect sites are classified into a direct synthesis strategy and postprocessing modification strategy. A direct synthesis strategy indicates constructing defects during the formation process of nanomaterials. Hydrothermal/solvothermal methods and the galvanic replacement method belong to this strategy. The postprocessing modification strategy, including chemical etching, physical etching and so on, means artificially creating defects after the formation of specific materials. Herein, we introduce typical defects engineering methods for point, line, surface and bulk volume defects, respectively. It should be mentioned that more than one kind of defects are usually formed in metal nanomaterials when defects are fabricated. Moreover, not only the synthesis process of nanomaterials, but also the catalytic CO_2_RR process can generate defects.

### 4.1. Point Defects Engineering

To date, a large number of synthetic methods have been reported in the literature for the generation of point defects, among which we will discuss the following typical examples, namely galvanic replacement and solvothermal methods.

**Figure 8:**
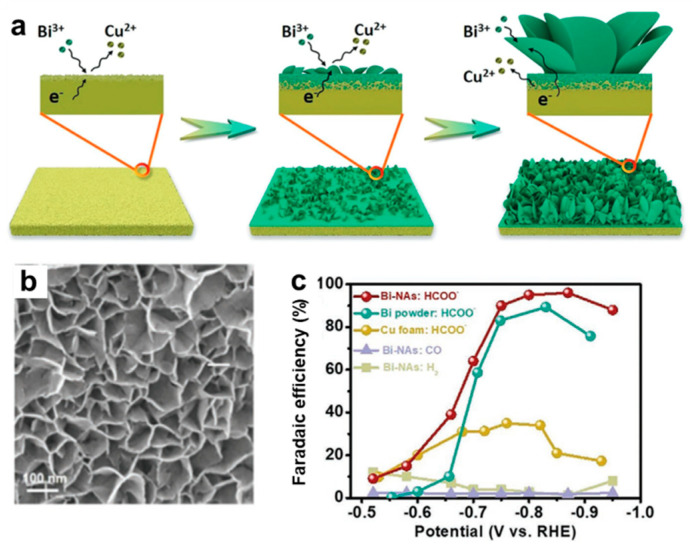
(**a**) Schematic diagram of the synthesis and (**b**) morphology of Bi nanosheets synthesized by galvanic replacement reaction; (**c**) Comparison of potential–dependent FE for HCOO^−^, CO, and H_2_ measured on Bi nanosheets (Bi–NAs), Bi powders and Cu foam. Reproduced with permission [71]. Copyright 2021, Wiley–VCH.

The classical galvanic replacement method has been widely used in the synthesis of various materials due to its mild conditions and easy operation process. The electrochemical catalysts with point defects towards CO_2_RR can be prepared as well. The galvanic replacement reaction takes advantage of the potential difference between two metals to achieve the purpose of atoms substitution, which is commonly known as the formation of one kind of metal by sacrificing another metal. In view of this, researchers have achieved the regulation of the composition of Cu–Pd by galvanic replacement reactions. By controlling the concentration of the Pd precursors, Cu–Pd products with different amounts of Pd dopants can be obtained. Among them, the Cu–Pd–0.3 (0.3% Pd) exhibited the highest FE toward alcohols generation from CO electroreduction because its *d*–band center is at the most suitable position, and the interaction between the two metals can better balance the adsorption of intermediate *HOCCH species and *H on the catalyst surface [36]. If the galvanic replacement reaction takes place more completely, alloys can be synthesized. Many reports concern the construction of catalysts towards CO_2_RR by this method. Li and co−workers used the galvanic replacement method to grow Bi nanosheet arrays on a Cu substrate (Figure 8a) [71]. These nanosheets were only 2–3 nm thick with a large surface area and abundant defects (Figure 8b). Due to the unique structure, the materials exhibited excellent catalytic activity with a high FE, high current density and good catalytic stability in the application of electrocatalytic CO_2_ to formate (Figure 8c).

The wet chemical reduction method, including the hydrothermal/solvothermal method, is one of the most widely studied synthesis methods. This method enables the synthesis of nanomaterials at relatively mild and green condition. Regulating the reaction parameters, such as the kind of reducing agent and the solvent, can lead to nanomaterials with different morphologies and surface structures. Both metal and nonmetal can dope into the nanomaterials for CO_2_RR. E.H. Sargent et al. modified the local electronic structure of Cu by doping nonmetal B [72]. The strong reducing agent NaBH_4_ was used. The concentration of B in the catalysts could be tuned by the amount CuCl_2_ precursor. Compared with the pristine copper and oxidized nanocopper catalysts, the B−doped Cu catalysts showed a more superior conversion efficiency, higher C_2_ selectivity and stability. Using the same synthetic method, halogene could be used to modify copper catalyst as well [73].

### 4.2. Line Defects Engineering

**Figure 9:**
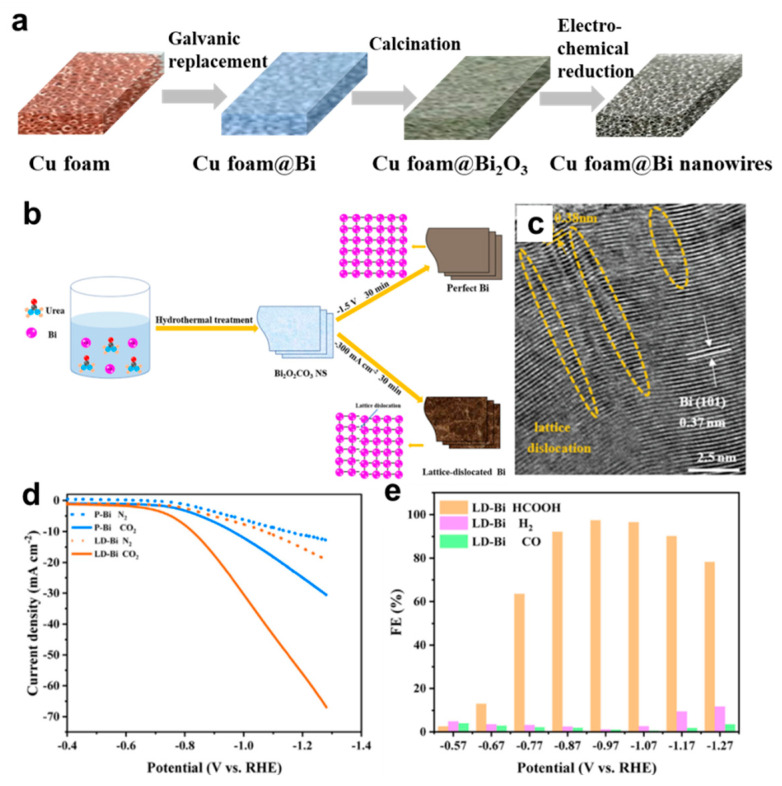
(**a**) Illustration of the synthesis of Cu foam@Bi nanowires. (**b**) Synthesis schematic and (**c**) HRTEM image for Bi nanosheets with rich dislocations. (**d**) Linear sweep voltammetry curves of perfect Bi (P-Bi) and lattice dislocated Bi nanosheets (LD-Bi) in a gas−saturated 0.5 M KHCO_3_ solution. (**e**) FE of HCOOH, H_2_, and CO for defective Bi nanosheets. Reproduced with permission [64]. Copyright 2022, Elsevier.

Line defects are always formed during the growth process of nanocrystals. So far, the successful construction of line defects in metal nanomaterials for application in CO_2_RR has rarely been reported. Zhang et al. reported the successful preparation of bismuth nanowires with rich dislocations by in situ electrochemical reduction of Bi_2_O_3_ that were previously coated on the surface of Cu foams (Figure 9a) [74]. A Bi_2_O_3_ layer on Cu foams was formed by calcinating the Bi layer, which was fabricated by the coordination−enabled galvanic replacement method. As bismuth has fragile mechanical properties, it is difficult to form porous Bi electrodes, thus porous Cu foams were used as templates. To achieve a uniform bismuth coating that was limited by the material transport in the electrolyte, ligands that could coordinate with Cu^+^ were used to form stable complexes, which increased the reduction potential difference between Cu^+^ and Bi^3+^ and prompted the replacement reaction to proceed rapidly. The lattice dislocation defects, which were generated during in situ electrochemical transformation or thermal treatment process, can be observed on a TEM image. Cu foam@Bi nanowires achieved an efficient conversion of CO_2_ to formic acid with an FE of 95% for formate at a potential of −0.69 V vs. RHE and they maintained a high activity in the potential range from −0.69 V to −0.99 V vs. RHE. The high CO_2_ reduction activity of the Cu foam@Bi nanowires electrode could be attributed to the lattice dislocation defects of the Bi nanowires and the large catalytic specific surface area due to their porous structure.

Ultrathin Bi nanosheets with abundant lattice dislocations were also reported, which were prepared by the topological transformation of Bi_2_O_2_CO_3_ nanosheets via electrochemical reduction at a current of −300 mA cm^−2^ (Figure 9b,c) [64]. Compared with perfect Bi nanosheets that were synthesized by reduction at low potential, the lattice–dislocation–rich Bi nanosheets showed a higher activity, selectivity and stability in converting CO_2_ to HCOOH (Figure 9d,e).

### 4.3. Surface Defects Engineering

As mentioned above, surface defects include surface step and boundary defects. Surface control to form surface step sites on nanomaterials has been widely studied since the first report of synthesizing high–index {720} facets–exposed Pt nanocrystals by Sun via an electrochemical method [75]. So far, many reviews have summarized the surface engineering results of metal nanocrystals for application in ORR and HER, especially noble metal nanocrystals. The surface control methods include the capping agent regulation, kinetics control, etching method, supersaturation control method, and so on [76,77,78,79]. As for constructing metal nanomaterials for CO_2_RR, these methods are still effective. We herein introduce the most typical methods.

**Figure 10:**
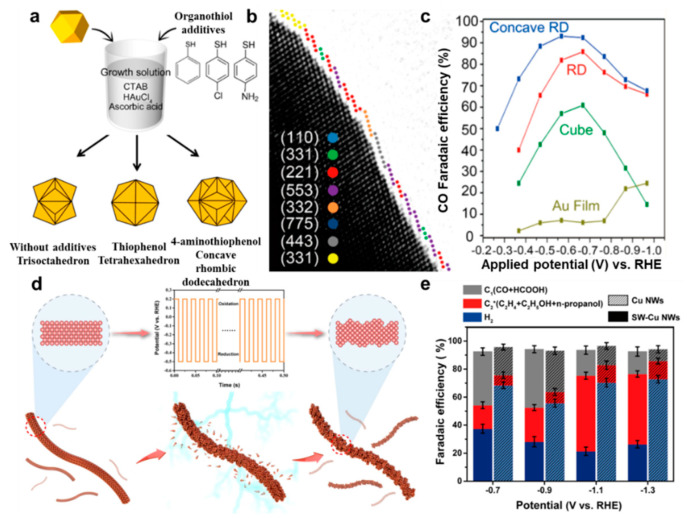
(**a**) Surface control of Au nanocrystals by capping agent. (**b**) HRTEM image showing the surface structure of a concave rhombic dodecahedral Au nanocrystal. (**c**) FE of CO for Au catalysts with shapes of film, cube, rhombic dodecahedron (RD) and concave rhombic dodecahedron. Reproduced with permission [80]. Copyright 2015, American Chemical Society. (**d**) Schematic illustration showing surface structure change of Cu nanowires by square–wave potential. (**e**) FE of products on perfect and square–wave potential treated–Cu nanowires (SW–Cu NWs). Reproduced with permission [81]. Copyright 2022, Wiley–VCH.

The surface control based on specific capping agents has a long history and is regarded as a relatively simple and practical method for the construction of targeted exposing facets. Although the intrinsic reason for surface control caused by the capping agent is not fully understood, the effect of specific capping agents on surface control could be summarized into two aspects. The specific adsorption between the capping agent and specific surface is one aspect, which alters the relative growth rates of various crystallographic planes and makes a specific surface thermodynamically favorable. The other aspect is that the configuration of a specific capping agent can affect the atoms deposition sites on the nucleus. Ki Tae Nam and co–workers reported the unique shape regulation of Au nanocrystals via a seeded–mediated method with the assistance of mercaptans [80]. The strength of thiol binding with Au is considered as the main reason that affects the final morphology. Without any additives, trisoctahedral Au nanocrystals are the final products (Figure 10a). The use of 4–aminothiophenol leads to the unique concave rhombic dodecahedral structure due to the stronger binding energy of −SH with Au in the presence of the –NH_2_ group, which has an electron−donating property. When in the presence of −H instead of –NH_2_ at the para–position, tetrahexahedral Au nanocrystals without obvious concave surface are the resultant nanocrystals, as interaction energy between −SH and the Au surface is slightly decreased. Apart from the thiol group, the configuration of capping agents also plays a vital role in forming this shape. By using a capping agent without benzene, nanocrystals with irregular shapes dominate the final products. The HRTEM image in Figure 10b shows the unique concave structure enclosed by various high indexes, such as the {331} facets, {221} facets, {553} facets and so on, which contain obvious surface steps. Due to the high density of high energy sites, the concave rhombic dodecahedral Au nanocrystals exhibited a selective conversion of CO_2_ to CO (Figure 10c).

The postprocessing method can be widely used to construct plane defects. Electrochemical processing is a typical method to drive the atoms rearrangement and the formation of plane defects during the oxidization, reduction or alternate oxidization and reduction process. For example, on the basis of Cu nanowires with a smooth surface of {100} facets, a square–wave potential treatment was conducted, making the surface atoms rearrangements during the alternate oxidization and reduction process (Figure 10d) [81]. After a long duration of treatment, the surface became very rough and stepped Cu {331} facets appeared. Compared to the plat {100} facet, the (311) high–index facet prompted the adsorption of the *COCOH intermediate and led to a high selectivity of the C_2+_ products, such as C_3_H_7_OH, C_2_H_2_ and C_2_H_5_OH (Figure 10e). Actually, catalysts could undergo reconstruction under the working condition, which might contribute to increasing the catalytic performance, as the intrinsic stable catalytic active sites could appear or increase. A study of the reconstruction process can reveal the intrinsic catalytic active sites. Recently, Qiao and co–workers studied the controllable reconstruction of Bi−MOFs to Bi nanosheets with surface defects for electrochemical CO_2_RR. The reconstruction process included the dissociation and conversion of Bi–MOF to Bi_2_O_2_CO_3_ through electrolyte mediation and reduction of Bi_2_O_2_CO_3_ to Bi by potential mediation [82]. The unsaturated Bi atoms that formed adjacent to the surface vacancies were regarded as active sites for adsorption of *OCHO intermediates and ultimately benefited to the production of formate.

**Figure 11:**
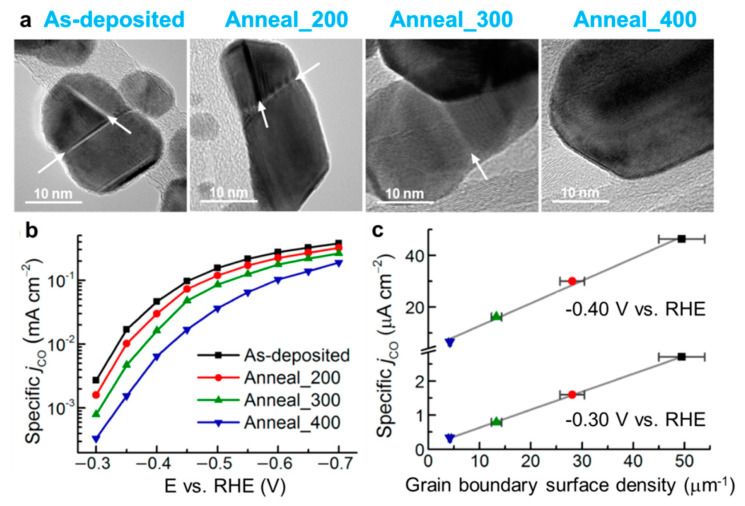
(**a**) TEM images of as−deposited and annealed Au/carbon nanotubes electrodes. (**b**) Specific current density for CO production (j_CO_) vs. potential and its (**c**) correlation with grain boundary surface density. Reproduced with permission [83]. Copyright 2015, American Chemical Society.

As for boundary engineering, the typical method to regulate the density is annealing. A higher temperature leads to a lower density of grain boundaries. M.W. Kanan and co−workers studied the quantitative correlation between grain boundaries density and catalytic activity towards CO_2_RR [83]. They prepared Au nanoparticles with rich grain boundaries on carbon nanotubes by the e–beam evaporation method and then tuned the density of grain boundaries by thermal annealing (Figure 11a). A higher annealing temperature decreased the boundaries density due to the decrease of total energy. With the increase of boundaries density, the current density of CO increased accordingly (Figure 11b). Moreover, they exhibited a linear relationship in the potential range from −0.3 V to −0.4 V for these four samples (Figure 11c). Except for the boundary engineering, there are many literature works reporting that the five–twinned structure exhibited superior performance towards CO_2_RR [45,84,85].

### 4.4. Volume Defects Engineering

Volume defects have three–dimensional structure that can be observed relatively more obviously and created more easily. Herein, we mainly introduce the template method and etching method.

**Figure 12:**
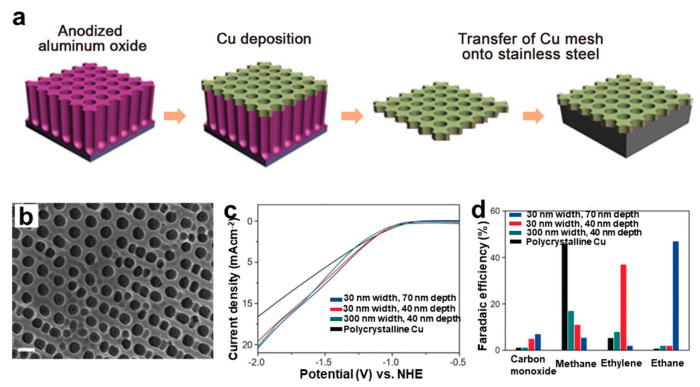
(**a**) Preparation scheme and (**b**) SEM images of copper mesoporous electrode. Scale = 300 nm. (**c**) Linear sweep voltammetry curves and (**d**) chemical selectivity at −1.7 V vs. normal hydrogen electrode (NHE) of four electrodes with different pore widths and depths. Reproduced with permission [87]. Copyright 2017, Wiley–VCH.

The template method can copy the template structure into the target material. This method has the advantages of a strong versatility and simple equipment. The templates can be diversifying. Gas can act as the template to create volume defects. For example, Sen et al. prepared 3D copper foam on a copper substrate using electrochemical deposition [86]. During deposition, H_2_ bubbles were used as templates to create pores, which could be tuned in the range of 20–50 μm. During the electrocatalytic CO_2_RR process, copper nanofoams contributed to the high selectivity of CO and HCOOH, which were significantly different from those obtained on the smooth electropolished copper electrode, indicating the defects effect caused by these different graded pores. Except for gas bubbles, anodized alumina usually serves as a typical template, in which a clear hole width and depth can be tuned, which is favorable to identify the actual active sites for catalytic regulation. Yang et al. designed and synthesized a mesoporous copper electrode that could accurately control the width and depth of the hole by sputtering copper on an anodized alumina template (Figure 12a,b) [87]. The results showed morphologically dependent product distributions (Figure 12c,d). The results indicated that a decrease in pore width and an increase in pore depth promoted the formation of C_2_ products.

**Figure 13:**
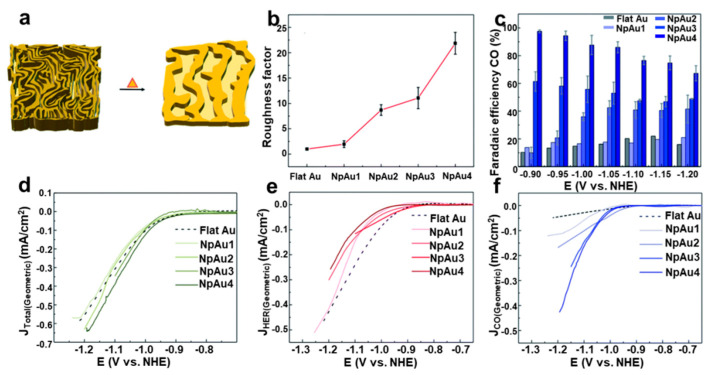
(**a**) Schematic diagram of nanoporous (Np) Au catalyst. (**b**) Roughness factors, (**c**) FE of CO, (**d**) total geometric current density, (**e**) partial geometric current densities of HER and (**f**) partial geometric current densities of CO over four porous Au catalysts. Reproduced with permission [88]. Copyright 2022, the Royal Society of Chemistry.

Etching is an important method to construct porous nanostructures by substitution, coordination or etching away unstable atoms. Akansha Goyal et al. studied the effect of pore diameter and pore length on regulating the CO_2_RR of nanoporous gold catalyst. Four different nanoporous gold catalysts with pore sizes ranging roughly from 40 nm to 10 nm were prepared by electrochemical alloying, dealloying and thermal coarsening (Figure 13a) [88]. The surface roughness factor of the catalysts decreased with the increase of thermal coarsening (Figure 13b). The FE of CO and the current density increased with the increase of the catalyst roughness factor (Figure 13c,d,f). The geometric current density of HER decreased from flat Au to Au with the increased roughness (Figure 13e). The results showed that the decrease of pore size and the increase of pore length increased the surface roughness of the catalyst, resulting in more surface defects, which led to more active sites, an enhanced CO_2_RR current, inhibited competitive HER reactions, and achieved a higher Faraday selectivity of CO that was close to 100%.

## 5. Conclusions and Perspectives

In conclusion, the research on developing metal nanomaterials with defects for electrocatalytic CO_2_RR was briefly summarized. Typical examples were illustrated to demonstrate the corresponding structure features and the characterization of metal nanocrystals with defects by EM techniques. In addition, defects engineering methods together with the recent progress related to electrocatalytic CO_2_RR were shown. For further contributions, future research directions for this field are proposed as follows: (1) Although there are many research works studying the effects of defects on metal nanomaterials towards CO_2_RR, the rational design and control of the amount and distribution of defects are rarely reported. The corresponding defects engineering strategies need to be developed. (2) So far, a limited understanding towards the growth mechanism of defects in nanomaterials has been demonstrated. Further research concerning an in–depth understanding by in situ spectroscopic and microscopic techniques is highly desired. (3) To achieve a long–term catalytic activity, the stability of defects during the catalytic performance should be paid attention to. In all, defects in nanomaterials are so vital and fascinating that they warrant devoting more efforts to exploring the construction of metal nanocrystals with defects.

## Data Availability

Not applicable.

## References

[1] Seneviratne S.I., Donat M.G., Pitman A.J., Knutti R., Wilby R.L. (2016). Allowable CO_2_ emissions based on regional and impact−related climate targets. Nature.

[2] Fan L., Xia C., Yang F., Wang J., Wang H., Lu Y. (2020). Strategies in catalysts and electrolyzer design for electrochemical CO_2_ reduction toward C_2+_ products. Sci. Adv..

[3] Gao W., Liang S., Wang R., Jiang Q., Zhang Y., Zheng Q., Xie B., Toe C.Y., Zhu X., Wang J. (2020). Industrial carbon dioxide capture and utilization: State of the art and future challenges. Chem. Soc. Rev..

[4] Al−Mamoori A., Krishnamurthy A., Rownaghi A.A., Rezaei F. (2017). Carbon Capture and Utilization Update. Energy Technol..

[5] Li K., Peng B., Peng T. (2016). Recent Advances in Heterogeneous Photocatalytic CO_2_ Conversion to Solar Fuels. ACS Catal..

[6] Zhu D.D., Liu J.L., Qiao S.Z. (2016). Recent Advances in Inorganic Heterogeneous Electrocatalysts for Reduction of Carbon Dioxide. Adv. Mater..

[7] Gallo A., Snider J.L., Sokaras D., Nordlund D., Kroll T., Ogasawara H., Kovarik L., Duyar M.S., Jaramillo T.F. (2020). Ni_5_Ga_3_ catalysts for CO_2_ reduction to methanol: Exploring the role of Ga surface oxidation/reduction on catalytic activity. Appl. Catal. B–Environ..

[8] Li M., Zhang L., Wu M., Du Y., Fan X., Wang M., Zhang L., Kong Q., Shi J. (2016). Mesostructured CeO_2_/g−C_3_N_4_ nanocomposites: Remarkably enhanced photocatalytic activity for CO_2_ reduction by mutual component activations. Nano Energy.

[9] Li Q., Wang S., Sun Z., Tang Q., Liu Y., Wang L., Wang H., Wu Z. (2019). Enhanced CH_4_ selectivity in CO_2_ photocatalytic reduction over carbon quantum dots decorated and oxygen doping g–C_3_N_4_. Nano Res..

[10] Wang H., Tzeng Y.K., Ji Y., Li Y., Li J., Zheng X., Yang A., Liu Y., Gong Y., Cai L. (2020). Synergistic enhancement of electrocatalytic CO_2_ reduction to C_2_ oxygenates at nitrogen−doped nanodiamonds/Cu interface. Nat. Nanotechnol..

[11] Kan M., Wang Q., Hao S., Guan A., Chen Y., Zhang Q., Han Q., Zheng G. (2022). System Engineering Enhances Photoelectrochemical CO_2_ Reduction. J. Phys. Chem. C.

[12] Cai S., Chen J., Li Q., Jia H. (2021). Enhanced Photocatalytic CO_2_ Reduction with Photothermal Effect by Cooperative Effect of Oxygen Vacancy and Au Cocatalyst. ACS Appl. Mater. Interfaces.

[13] Li Z., Zhang L., Huang W., Xu C., Zhang Y. (2021). Photothermal Catalysis for Selective CO_2_ Reduction on the Modified Anatase TiO_2_ (101) Surface. ACS Appl. Energy Mater..

[14] Ross M.B., De Luna P., Li Y., Dinh C.-T., Kim D., Yang P., Sargent E.H. (2019). Designing materials for electrochemical carbon dioxide recycling. Nat. Catal..

[15] Shao Q., Wang P., Zhu T., Huang X. (2019). Low Dimensional Platinum–Based Bimetallic Nanostructures for Advanced Catalysis. Acc. Chem. Res..

[16] Sarkar S., Peter S.C. (2018). An overview on Pd–based electrocatalysts for the hydrogen evolution reaction. Inorg. Chem. Front..

[17] Xie H., Wang T., Liang J., Li Q., Sun S. (2018). Cu–based nanocatalysts for electrochemical reduction of CO_2_. Nano Today.

[18] Chen Y., Li C.W., Kanan M.W. (2012). Aqueous CO_2_ reduction at very low overpotential on oxide–derived Au nanoparticles. J. Am. Chem. Soc..

[19] Shi R., Guo J., Zhang X., Waterhouse G.I.N., Han Z., Zhao Y., Shang L., Zhou C., Jiang L., Zhang T. (2020). Efficient wettability–controlled electroreduction of CO_2_ to CO at Au/C interfaces. Nat. Commun..

[20] Zhang N., Zhang X., Tao L., Jiang P., Ye C., Lin R., Huang Z., Li A., Pang D., Yan H. (2021). Silver Single–Atom Catalyst for Efficient Electrochemical CO_2_ Reduction Synthesized from Thermal Transformation and Surface Reconstruction. Angew. Chem. Int. Ed..

[21] Gao D., Zhou H., Wang J., Miao S., Yang F., Wang G., Wang J., Bao X. (2015). Size–dependent electrocatalytic reduction of CO_2_ over Pd nanoparticles. J. Am. Chem. Soc..

[22] Zhao S., Li S., Guo T., Zhang S., Wang J., Wu Y., Chen Y. (2019). Advances in Sn–Based Catalysts for Electrochemical CO_2_ Reduction. Nanomicro. Lett..

[23] Zhang W., Hu Y., Ma L., Zhu G., Zhao P., Xue X., Chen R., Yang S., Ma J., Liu J. (2018). Liquid−phase exfoliated ultrathin Bi nanosheets: Uncovering the origins of enhanced electrocatalytic CO_2_ reduction on two–dimensional metal nanostructure. Nano Energy.

[24] Li W., Wang D., Zhang Y., Tao L., Wang T., Zou Y., Wang Y., Chen R., Wang S. (2020). Defect Engineering for Fuel–Cell Electrocatalysts. Adv. Mater..

[25] Zhang Y., Guo L., Tao L., Lu Y., Wang S. (2018). Defect–Based Single–Atom Electrocatalysts. Small Methods.

[26] Yan D., Li H., Chen C., Zou Y., Wang S. (2018). Defect Engineering Strategies for Nitrogen Reduction Reactions under Ambient Conditions. Small Methods.

[27] Yan D., Li Y., Huo J., Chen R., Dai L., Wang S. (2017). Defect Chemistry of Nonprecious−Metal Electrocatalysts for Oxygen Reactions. Adv. Mater..

[28] Jia Y., Jiang K., Wang H., Yao X. (2019). The Role of Defect Sites in Nanomaterials for Electrocatalytic Energy Conversion. Chem.

[29] Xie C., Yan D., Chen W., Zou Y., Chen R., Zang S., Wang Y., Yao X., Wang S. (2019). Insight into the design of defect electrocatalysts: From electronic structure to adsorption energy. Mater. Today.

[30] Li G., Blake G.R., Palstra T.T. (2017). Vacancies in functional materials for clean energy storage and harvesting: The perfect imperfection. Chem. Soc. Rev..

[31] Zhu W., Zhang L., Yang P., Hu C., Dong H., Zhao Z.-T., Mu R., Gong J. (2018). Formation of Enriched Vacancies for Enhanced CO_2_ Electrocatalytic Reduction over AuCu Alloys. ACS Energy Lett..

[32] Kotakoski J., Mangler C., Meyer J.C. (2014). Imaging atomic-level random walk of a point defect in graphene. Nat. Commun..

[33] Jiang Y., Chen Z., Han Y., Deb P., Gao H., Xie S., Purohit P., Tate M.W., Park J., Gruner S.M. (2018). Electron ptychography of 2D materials to deep sub–angstrom resolution. Nature.

[34] Van Benthem K., Lupini A.R., Kim M., Baik H.S., Doh S., Lee J.-H., Oxley M.P., Findlay S.D., Allen L.J., Luck J.T. (2005). Three–dimensional imaging of individual hafnium atoms inside a semiconductor device. Appl. Phys. Lett..

[35] Zhu Y., Tao L., Chen X., Ma Y., Ning S., Zhou J., Zhao X., Bosman M., Liu Z., Du S. (2021). Anisotropic point defects in rhenium diselenide monolayers. iScience.

[36] Li J., Xu A., Li F., Wang Z., Zou C., Gabardo C.M., Wang Y., Ozden A., Xu Y., Nam D.H. (2020). Enhanced multi–carbon alcohol electroproduction from CO via modulated hydrogen adsorption. Nat. Commun..

[37] Zhang Z., Liu G., Cui X., Gong Y., Yi D., Zhang Q., Zhu C., Saleem F., Chen B., Lai Z. (2021). Evoking ordered vacancies in metallic nanostructures toward a vacated Barlow packing for high–performance hydrogen evolution. Sci. Adv..

[38] Shao W., Pan Q., Chen Q., Zhu C., Tao W., Zhu H., Song H., Liu X., Tan P.H., Sheng G. (2020). Symmetry Breaking in Monometallic Nanocrystals toward Broadband and Direct Electron Transfer Enhanced Plasmonic Photocatalysis. Adv. Funct. Mater..

[39] Zhou S., Zhao M., Yang T.-H., Xia Y. (2019). Decahedral nanocrystals of noble metals: Synthesis, characterization, and applications. Mater. Today.

[40] Wang H., Zhou S., Gilroy K.D., Cai Z., Xia Y. (2017). Icosahedral nanocrystals of noble metals: Synthesis and applications. Nano Today.

[41] Lee S.R., Vara M., Hood Z.D., Zhao M., Gilroy K.D., Chi M., Xia Y. (2018). Rhodium Decahedral Nanocrystals: Facile Synthesis, Mechanistic Insights, and Experimental Controls. ChemNanoMat.

[42] Tang Y., Edelmann R.E., Zou S. (2014). Length tunable penta–twinned palladium nanorods: Seedless synthesis and electrooxidation of formic acid. Nanoscale.

[43] Gao Y., Jiang P., Song L., Wang J.X., Liu L.F., Liu D.F., Xiang Y.J., Zhang Z.X., Zhao X.W., Dou X.Y. (2006). Studies on silver nanodecahedrons synthesized by PVP–assisted N,N–dimethylformamide (DMF) reduction. J. Crys. Growth.

[44] Song M., Wu Z., Lu N., Li D. (2019). Strain Relaxation-Induced Twin Interface Migration and Morphology Evolution of Silver Nanoparticles. Chem. Mater..

[45] Choi C., Cheng T., Flores Espinosa M., Fei H., Duan X., Goddard W.A., Huang Y. (2019). A Highly Active Star Decahedron Cu Nanocatalyst for Hydrocarbon Production at Low Overpotentials. Adv. Mater..

[46] Johnson C.L., Snoeck E., Ezcurdia M., Rodriguez-Gonzalez B., Pastoriza-Santos I., Liz-Marzan L.M., Hytch M.J. (2008). Effects of elastic anisotropy on strain distributions in decahedral gold nanoparticles. Nat. Mater..

[47] Chatterjee D., Shetty S., Muller-Caspary K., Grieb T., Krause F.F., Schowalter M., Rosenauer A., Ravishankar N. (2018). Ultrathin Au–Alloy Nanowires at the Liquid–Liquid Interface. Nano Lett..

[48] Yu Y., Cui F., Sun J., Yang P. (2016). Atomic Structure of Ultrathin Gold Nanowires. Nano Lett..

[49] Roy A., Kundu S., Muller K., Rosenauer A., Singh S., Pant P., Gururajan M.P., Kumar P., Weissmuller J., Singh A.K. (2014). Wrinkling of atomic planes in ultrathin Au nanowires. Nano Lett..

[50] Wang C., Zhang Z., Yang G., Chen Q., Yin Y., Jin M. (2016). Creation of Controllable High–Density Defects in Silver Nanowires for Enhanced Catalytic Property. Nano Lett..

[51] Fan Z., Bosman M., Huang X., Huang D., Yu Y., Ong K.P., Akimov Y.A., Wu L., Li B., Wu J. (2015). Stabilization of 4H hexagonal phase in gold nanoribbons. Nat. Commun..

[52] Chen Y., Fan Z., Luo Z., Liu X., Lai Z., Li B., Zong Y., Gu L., Zhang H. (2017). High–Yield Synthesis of Crystal-Phase–Heterostructured 4H/fcc Au@Pd Core–Shell Nanorods for Electrocatalytic Ethanol Oxidation. Adv. Mater..

[53] Zhang N., Bu L., Guo S., Guo J., Huang X. (2016). Screw Thread–Like Platinum–Copper Nanowires Bounded with High–Index Facets for Efficient Electrocatalysis. Nano Lett..

[54] Zhang F.Y., Sheng T., Tian N., Liu L., Xiao C., Lu B.A., Xu B.B., Zhou Z.Y., Sun S.G. (2017). Cu overlayers on tetrahexahedral Pd nanocrystals with high–index facets for CO_2_ electroreduction to alcohols. Chem. Commun..

[55] Koh J.H., Won D.H., Eom T., Kim N.-K., Jung K.D., Kim H., Hwang Y.J., Min B.K. (2017). Facile CO_2_ Electro-Reduction to Formate via Oxygen Bidentate Intermediate Stabilized by High–Index Planes of Bi Dendrite Catalyst. ACS Catal..

[56] Rosen J., Hutchings G.S., Lu Q., Rivera S., Zhou Y., Vlachos D.G., Jiao F. (2015). Mechanistic Insights into the Electrochemical Reduction of CO_2_ to CO on Nanostructured Ag Surfaces. ACS Catal..

[57] Huo H., Wang J., Fan Q., Hu Y., Yang J. (2021). Cu–MOFs Derived Porous Cu Nanoribbons with Strengthened Electric Field for Selective CO_2_ Electroreduction to C_2+_ Fuels. Adv. Energy Mater..

[58] Wang Y., Li Y., Liu J., Dong C., Xiao C., Cheng L., Jiang H., Jiang H., Li C. (2021). BiPO_4_–Derived 2D Nanosheets for Efficient Electrocatalytic Reduction of CO_2_ to Liquid Fuel. Angew. Chem. Int. Ed..

[59] Nguyen D.L.T., Kim Y., Hwang Y.J., Won D.H. (2019). Progress in development of electrocatalyst for CO_2_ conversion to selective CO production. Carbon Energy.

[60] Zhang L., Li X.X., Lang Z.L., Liu Y., Liu J., Yuan L., Lu W.Y., Xia Y.S., Dong L.Z., Yuan D.Q. (2021). Enhanced Cuprophilic Interactions in Crystalline Catalysts Facilitate the Highly Selective Electroreduction of CO_2_ to CH_4_. J. Am. Chem. Soc..

[61] Zhang B., Zhang J., Hua M., Wan Q., Su Z., Tan X., Liu L., Zhang F., Chen G., Tan D. (2020). Highly Electrocatalytic Ethylene Production from CO_2_ on Nanodefective Cu Nanosheets. J. Am. Chem. Soc..

[62] Jiao S., Fu X., Zhang L., Zeng Y.-J., Huang H. (2020). Point–defect–optimized electron distribution for enhanced electrocatalysis: Towards the perfection of the imperfections. Nano Today.

[63] Wu D., Feng R., Xu C., Sui P.F., Zhang J., Fu X.Z., Luo J.L. (2021). Regulating the Electron Localization of Metallic Bismuth for Boosting CO_2_ Electroreduction. Nanomicro Lett..

[64] Wang Y., Gong H., Wang Y., Gao L. (2022). Lattice-dislocated Bi nanosheets for electrocatalytic reduction of carbon dioxide to formate over a wide potential window. J. Colloid Interf. Sci..

[65] Li C.W., Ciston J., Kanan M.W. (2014). Electroreduction of carbon monoxide to liquid fuel on oxide–derived nanocrystalline copper. Nature.

[66] Cheng T., Huang Y., Xiao H., Goddard W.A. (2017). Predicted Structures of the Active Sites Responsible for the Improved Reduction of Carbon Dioxide by Gold Nanoparticles. J. Phys. Chem. Lett..

[67] Dong C., Fu J., Liu H., Ling T., Yang J., Qiao S.Z., Du X.-W. (2017). Tuning the selectivity and activity of Au catalysts for carbon dioxide electroreduction via grain boundary engineering: A DFT study. J. Mater. Chem. A.

[68] Hu F., Abeyweera S.C., Yu J., Zhang D., Wang Y., Yan Q., Sun Y. (2020). Quantifying Electrocatalytic Reduction of CO_2_ on Twin Boundaries. Chem.

[69] Luo W., Zhang J., Li M., Züttel A. (2019). Boosting CO Production in Electrocatalytic CO_2_ Reduction on Highly Porous Zn Catalysts. ACS Catal..

[70] Wang J., Zou J., Hu X., Ning S., Wang X., Kang X., Chen S. (2019). Heterostructured intermetallic CuSn catalysts: High performance towards the electrochemical reduction of CO_2_ to formate. J. Mater. Chem. A.

[71] Fan J., Zhao X., Mao X., Xu J., Han N., Yang H., Pan B., Li Y., Wang L., Li Y. (2021). Large–Area Vertically Aligned Bismuthene Nanosheet Arrays from Galvanic Replacement Reaction for Efficient Electrochemical CO_2_ Conversion. Adv. Mater..

[72] Zhou Y., Che F., Liu M., Zou C., Liang Z., De Luna P., Yuan H., Li J., Wang Z., Xie H. (2018). Dopant–induced electron localization drives CO_2_ reduction to C_2_ hydrocarbons. Nat. Chem..

[73] Li M., Ma Y., Chen J., Lawrence R., Luo W., Sacchi M., Jiang W., Yang J. (2021). Residual Chlorine Induced Cationic Active Species on a Porous Copper Electrocatalyst for Highly Stable Electrochemical CO_2_ Reduction to C_2_. Angew. Chem. Int. Ed..

[74] Zhang X., Sun X., Guo S.-X., Bond A.M., Zhang J. (2019). Formation of lattice–dislocated bismuth nanowires on copper foam for enhanced electrocatalytic CO_2_ reduction at low overpotential. Energy Environ. Sci..

[75] Tian N., Zhou Z.Y., Sun S.G., Ding Y., Wang Z.L. (2007). Synthesis of tetrahexahedral platinum nanocrystals with high–index facets and high electro–oxidation activity. Science.

[76] Lin H.X., Lei Z.C., Jiang Z.Y., Hou C.P., Liu D.Y., Xu M.M., Tian Z.Q., Xie Z.X. (2013). Supersaturation–dependent surface structure evolution: From ionic, molecular to metallic micro/nanocrystals. J. Am. Chem. Soc..

[77] Xie S., Zhang H., Lu N., Jin M., Wang J., Kim M.J., Xie Z., Xia Y. (2013). Synthesis of rhodium concave tetrahedrons by collectively manipulating the reduction kinetics, facet–selective capping, and surface diffusion. Nano Lett..

[78] Zhang Z.C., Nosheen F., Zhang J.C., Yang Y., Wang P.P., Zhuang J., Wang X. (2013). Growth of concave polyhedral Pd nanocrystals with 32 facets through in situ facet–selective etching. ChemSusChem.

[79] Jia Y., Jiang Y., Zhang J., Zhang L., Chen Q., Xie Z., Zheng L. (2014). Unique excavated rhombic dodecahedral PtCu_3_ alloy nanocrystals constructed with ultrathin nanosheets of high–energy {110} facets. J. Am. Chem. Soc..

[80] Lee H.E., Yang K.D., Yoon S.M., Ahn H.Y., Lee Y.Y., Chang H., Jeong D.H., Lee Y.S., Kim M.Y., Nam K.T. (2015). Concave Rhombic Dodecahedral Au Nanocatalyst with Multiple High–Index Facets for CO_2_ Reduction. ACS Nano.

[81] Han L., Tian B., Gao X., Zhong Y., Wang S., Song S., Wang Z., Zhang Y., Kuang Y., Sun X. (2022). Copper nanowire with enriched high–index facets for highly selective CO_2_ reduction. SmartMat.

[82] Yao D., Tang C., Vasileff A., Zhi X., Jiao Y., Qiao S.Z. (2021). The Controllable Reconstruction of Bi–MOFs for Electrochemical CO_2_ Reduction through Electrolyte and Potential Mediation. Angew. Chem. Int. Ed..

[83] Feng X., Jiang K., Fan S., Kanan M.W. (2015). Grain–boundary–dependent CO_2_ electroreduction activity. J. Am. Chem. Soc..

[84] Huang H., Jia H., Liu Z., Gao P., Zhao J., Luo Z., Yang J., Zeng J. (2017). Understanding of Strain Effects in the Electrochemical Reduction of CO_2_: Using Pd Nanostructures as an Ideal Platform. Angew. Chem. Int. Ed..

[85] Lyu Z., Zhu S., Xu L., Chen Z., Zhang Y., Xie M., Li T., Zhou S., Liu J., Chi M. (2021). Kinetically Controlled Synthesis of Pd-Cu Janus Nanocrystals with Enriched Surface Structures and Enhanced Catalytic Activities toward CO_2_ Reduction. J. Am. Chem. Soc..

[86] Sen S., Liu D., Palmore G.T.R. (2014). Electrochemical Reduction of CO_2_ at Copper Nanofoams. ACS Catal..

[87] Yang K.D., Ko W.R., Lee J.H., Kim S.J., Lee H., Lee M.H., Nam K.T. (2017). Morphology–Directed Selective Production of Ethylene or Ethane from CO_2_ on a Cu Mesopore Electrode. Angew. Chem. Int. Ed..

[88] Goyal A., Bondue C.J., Graf M., Koper M.T.M. (2022). Effect of pore diameter and length on electrochemical CO_2_ reduction reaction at nanoporous gold catalysts. Chem. Sci..

